# A Competition-Based Problem-Posing Approach for Nursing Training

**DOI:** 10.3390/healthcare10061132

**Published:** 2022-06-17

**Authors:** Han-Yu Sung

**Affiliations:** Department of Allied Health Education and Digital Learning, National Taipei University of Nursing and Health Sciences, Taipei 11219, Taiwan; hanyu.sung@gmail.com; Tel.: +886-2-2822-7101 (ext. 3673)

**Keywords:** problem-posing strategy, competition, learning patterns, cognitive load

## Abstract

Conventional nursing teaching usually adopts one-way teaching approaches. As such, students cannot think deeply and engage in learning, which results in lower learning motivation and learning achievement. Several studies have indicated that problem-posing is a learning process that has students think about problems and actively construct knowledge, which helps their in-depth thinking and promotes their learning achievement. However, problem-posing is a task with a higher difficulty level; in particular, with insufficient learning motivation, it is not easy for students to pose in-depth questions. Therefore, the present study introduced competition to a problem-posing activity to facilitate students’ motivation. This study adopted a quasi-experimental design and conducted an experiment in the unit of Care of Critically Ill Patients. The results showed that the proposed competition-based problem-posing mobile learning approach could significantly enhance students’ learning achievement and learning motivation and would not cause an excessive cognitive load. Moreover, competition increased students’ learning motivation, and fostered them to actively reflect on and revise their questions, thereby increasing their problem-posing quality and learning achievement. This study can serve as a reference for future clinical practice to enhance the quality and sustainability of apprenticeships.

## 1. Introduction

Care of critically ill patients is an important training course in major medical care institutions and related schools in Taiwan. Since basic life support (BLS) has been corroborated as having a positive effect on the recovery of spontaneous circulation (ROSC) and survival to discharge, it is regarded as a basic first aid procedure and a skill that nurses should possess. All the critical care personnel in major medical institutions in Taiwan must hold this certificate. Relevant training courses are also regularly held in domestic medical institutions and schools; the purpose of such courses is to enable learners to make accurate judgements and provide appropriate treatment when encountering unknown emergencies. Studies have pointed out that the knowledge retention after first-aid training is limited. There is a decline in nurses’ first aid knowledge and skills within one year after the training. First aid training held every two to three years is not sufficient for nurses to master first aid procedures and the relevant crucial knowledge. In addition, the course content only focuses on the practice of first aid procedures instead of providing in-depth lectures on first aid knowledge. In first aid situations, nurses with less first aid knowledge tend to feel stressed [1,2]. International statistics further show that effective first aid training is the key to reducing nursing staff’s stress in the workplace and to increasing the success of emergency treatment [3,4].

In conventional teaching, due to the limited time, inflexible pace of teaching, and labor costs, teachers usually provide all the students with the same learning materials, adopt identical teaching approaches, and teach at the same pace. After a unit is completed, students are given paper-and-pencil tests or learning sheets to test their learning. Therefore, it is difficult to consider students’ individual learning needs. Students with a higher proficiency level are easily limited by a teacher’s teaching pace such that they cannot effectively learn in class. On the other hand, due to the restrictions of the course schedule, students with a lower proficiency level may be forced to learn the next unit without comprehending the course content, which results in ineffective learning and low learning motivation. As a response, using a problem-posing activity, the present study aimed to provide students with more opportunities for thinking and interaction, thereby fostering their reflection. In general, students have more involvement in solving self-posed problems than in solving questions in textbooks or raised by teachers. In other words, if a problem is posed by the problem solver, he/she will have higher motivation to solve it, as indicated by several researchers [5].

In several learning activities related to higher-order thinking, the strategy of guiding students to think deeply and organize knowledge through problem-posing has aroused much attention from educators and has become one of the important issues in technological learning [6,7,8]. For example, some researchers have embedded problem-posing tasks in learning sheets and found them effective in terms of improving nursing students’ learning outcomes [9,10]. Researchers have indicated that problem-posing learning activities can help students to construct knowledge, develop higher-order thinking, and cultivate their ability to analyze knowledge and develop problems [11,12,13]. However, previous research also pointed out that in comparison with problem-solving, problem-posing is a more difficult task [14]. Without sufficient and appropriate encouragement and guidance, students’ willingness and performance in problem-posing activities could be significantly affected [15,16,17]. Hence, it is an extremely important and difficult issue to promote students’ involvement in problem-posing activities with proper guidance [18].

Previous research has shown that competition could assist students in enhancing their learning achievement through interaction in the learning process [19,20]. A competitive learning approach means that learners achieve the learning objectives through the competition process and its results [21]. Competition among student groups will both motivate and help them to participate in order to achieve the goals of the competition. Furthermore, their involvement in the competition prompts students to participate in group discussions and to try to obtain answers from the internet or books, thereby helping them improve their problem-solving and critical thinking skills [22].

Past studies have verified that competition will make students feel excited and more positive when solving challenging problems in real-life situations [23,24]. Admiraal et al. (2011) found that students were affected by the scoring and reward mechanism of a game, and then competed with peers in the gaming process. It was found that the more students engaged in the competition, the more knowledge they acquired [19]. That is to say, students were constantly learning new information during the competition. To ensure that their answers or decisions are correct, students will be more cautious in the competition process [25]. In addition, competition can not only help students learn, but can also improve their learning motivation [26,27,28].

As a result, the present study developed a competition-based problem-posing mobile learning approach and applied it to the training for care of critically ill patients. The proposed approach guided students to be familiar with the procedures and relevant crucial knowledge about care of critically ill patients, aiming to promote nursing students’ learning achievement and learning motivation, as well as to reduce their cognitive load. Moreover, this study also explored students’ cognitive process and learning behaviors through a learning log analysis, which can serve as a reference for future educational institutions to plan technological learning strategies and objectives. The research questions are as follows:

1. Are there any significant differences between the learning achievement of students who adopt the competition-based problem-posing approach and the conventional problem-posing approach?
2. Are there any significant differences between the learning motivation of students who adopt the competition-based problem-posing approach and the conventional problem-posing approach?
3. What are the effects of the competition-based problem-posing approach on students’ learning behaviors?

## 2. Literature Review

### 2.1. Competition

Competition refers to the actions of comparing one’s own performances or confronting others who have identical goals [29]. Based on the social interdependence theory, group competition occurs when one group works cooperatively to compete with other groups [30]. The elements of competition include scores or a leaderboard. Scores can assist students in evaluating their performances [31], while a leaderboard, a common mechanism, enhances students’ learning motivation and enables them to view their learning progress immediately [32,33]. The competition contexts induce competition which may improve students’ learning performance and learning motivation [24]. Moreover, previous studies have specified that especially in competition, students will be stimulated by external environmental factors in the learning environment, thereby influencing the degree and speed of students’ individual growth in knowledge [34].

Educational competitions have been verified as an effective approach as they can increase students’ learning performances and learning motivation [23,24]. They have also been applied in various disciplines or contexts, for example, language learning [20], history [19], business [24], computer programming, and physical education [35]. Numerous studies have disclosed that educational competition can not only facilitate students’ learning [16,30], but can also enhance their social skills [28,36,37].

Competition is the process and behaviors whereby many people work hard for the same goal at the same time [38]. In the process of constructive competition, it is also a process of knowledge conflicts. Students strive to accomplish their goals and learning tasks, and both losers and winners obtain rewards. For instance, Cagiltay et al. (2015) proposed an educational competition game and trained university students to be able to respond when a database was attacked. The results showed that students not only enhanced their learning achievement, but also increased their learning motivation and engagement in the learning process [26]. Besides, the winners acquired knowledge and taught the losers how to modify and improve, making the competition more pleasurable and potentially valuable.

In addition, Deutsch et al. (2013) developed an online competition-based game which entailed learning Chinese characters to explore students’ cognitive load and competition anxiety [38]. A total of 220 sixth graders were recruited in this experiment. The findings revealed that even though students had a higher cognitive load and competition anxiety, they still held positive attitudes and intentions towards participation in the game. Nonetheless, students with higher cognitive load might lose their motivation to accomplish the challenges in the game [38]. Wang (2015) pinpointed that such variables as course content and peer competitiveness should be controlled appropriately. It made students lose motivation for competition or class due to noticeable disparities resulting from overly simple learning content or differences in students’ abilities [39]. Therefore, it is worth exploring how to effectively integrate competition into the educational learning environment so as to enhance students’ learning motivation and reduce their cognitive load.

### 2.2. Problem-Posing

Since the 1980s, the core values of problem-posing strategies have received attention in mathematics teaching [5]. Lin and Leng (2008) believed that problem-posing and problem-solving were concurrent [40]. Polya (1945) indicated that problem-solving consisted of four phases: Understand, Plan, Carry Out, and Look Back [41]. Before problem-solving, a person must comprehend the problem, consider how to solve it, and then take action. Tsubota (1987) disclosed that problem-posing was to pose new problems from the problem-solving process; a teacher gave students problems and asked them to answer first, and then students created new problems based on the experiences and knowledge gained in the problem-solving process [42]. Leung (1993) pointed out that problem-posing was related to problem-solving to a certain extent and proposed four phases of problem-posing based on Polya (1945): Pose, Plan, Carry Out, and Look Back. The problem poser also plays the role of the problem solver, so there is no need to go through the phase of “understanding” [41,43]. A person directly poses problems, plans and carries out problem-solving, generates new problems when looking back at the problem-solving process, and poses problems again, thus forming a cycle [43].

Problem-posing is an active, constructive learning process in which individuals organize knowledge and think about problems [7]. Through problem-posing, students can reflect on whether these problems are truly appropriate. No matter whether problem-posing or problem-solving, students are required to identify the key to the problem, figure out a solution, and carry out reflection, so as to cultivate their problem-solving skills. Several studies have corroborated that problem-posing enables students to think actively and improve their learning achievement. Students’ interactions in group discussions enhance their learning motivation and cultivates their thinking, analytical, and problem-solving abilities [44,45]. Researchers have also pointed out that students must pose thought-provoking problems; it is more difficult to cultivate students’ higher-order thinking through recitation problems [16,46]. Silver and Cai (2005) also indicated that problem-posing is another assessment method. Instructors can understand students’ learning status through their problem-posing [7]. For the mathematics curriculum and evaluation standards, the National Council of Teachers of Mathematics in the United States recommends that students should be allowed to engage in problem-posing activities through experiences, perceptions, and problem formation in mathematics classrooms as the focus of mathematics education, in order to increase their interest in problem-solving.

Chang et al. (2012) explored the effects of different problem-posing strategies on elementary school students’ problem-posing in mathematics. The results showed that the students adopting the online problem-posing strategy had better problem-posing ability, problem-solving ability, and flow experience than those adopting the conventional paper-based problem-posing strategy [16]. Arikan and Ünal (2015) investigated a problem-posing creative activity in mathematics education for eighth graders. The findings revealed that the experimental group and the control group had different problem-solving abilities, but not problem-posing abilities [47]. In addition, researchers have pointed out that problem-posing ability is affected by other factors such as learning motivation. Relevant research has pinpointed that engaging students in a problem-posing activity for knowledge construction has great potential for improving their learning performances, motivation, attitudes, and even peer interaction, problem analysis, and problem-solving skills [14,44,48]. Numerous researchers have further specified that the integration of technology and problem-posing strategies is conducive to promoting learners’ learning achievement [8,11,12]. Hence, the present study attempted to develop an effective problem-posing guidance and reward mechanism in a competition-based mobile learning approach, so as to improve students’ learning achievement and learning motivation.

## 3. Methods

### 3.1. Development of a Competition-Based Problem-Posing Learning Environment

In this study, a competition-based problem-posing mobile learning approach was developed. Through the competitive learning environment, individual problem-posing-based learning scaffolding was provided to facilitate students’ learning achievement and learning motivation and reduce their cognitive load.

**Figure 1:**
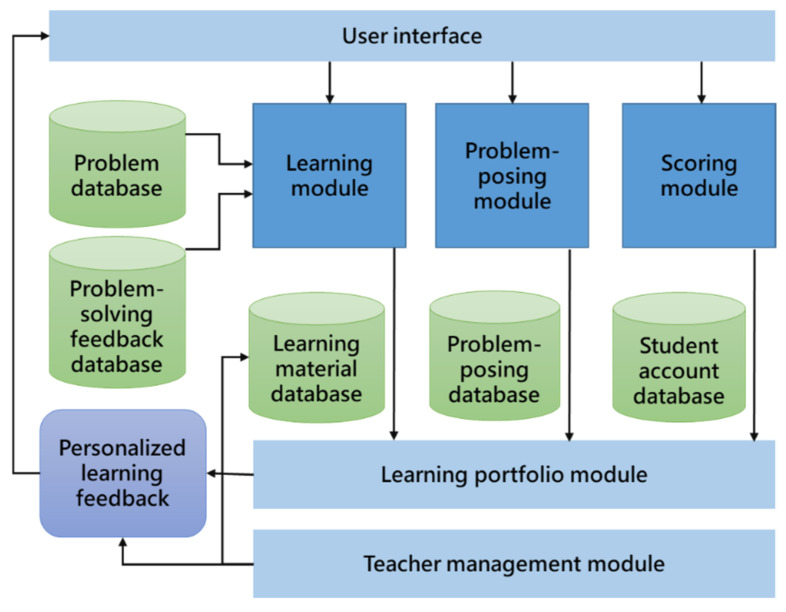
System structure.

In the competition-based problem-posing mobile learning environment, a “competition-based problem-posing feedback subsystem” guided students to accomplish the problem-posing competition in the system to increase the quality of their problem-posing and learning achievement. A “competition-based problem-solving feedback subsystem” enhanced students’ higher-order thinking through problem-solving competition in the game, to facilitate their learning achievement and learning motivation. Through a “competition-based problem-posing management subsystem,” the teacher was allowed to view the results of students’ problem-posing, problem-solving, and scores at any time. Understanding the quality of students’ problem-posing instantly helped students achieve effective learning. Moreover, a “learning portfolio and management subsystem” was used to record and analyze students’ learning behaviors to further explore the factors affecting learning achievement, as a reference for the subsequent improvement of learning content and activities. In addition to these subsystems, multiple databases were also developed, including a problem-posing database, a student account database, a learning material database, and a learning portfolio database. Figure 1 shows the system structure.

**Figure 2:**
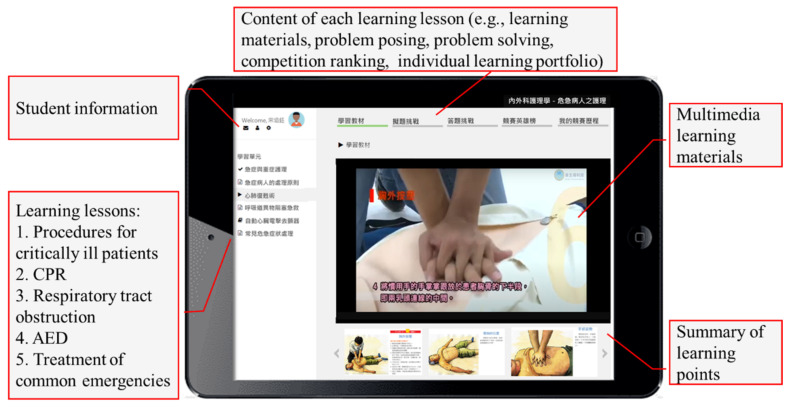
The competition-based problem-posing mobile learning system.

Problem-posing learning activities enable students to construct knowledge and develop higher-order thinking skills actively. According to the Zone of Proximal Development (ZPD) introduced by Vygotsky (1978), teachers can assist learners in reaching their potential development [49]. Skinner (1991) emphasized that problems used in class must be self-posed and thought-provoking [46]. On the contrary, easy problems cannot produce the effect of problem-posing teaching. During the class, a teacher skillfully asks students to pose problems and gives them the opportunity to make revisions. When students solve problems posed by others, they may ask the problem poser questions; the problem poser can then identify the errors in their self-posed problem and revise accordingly. This not only helps others understand the meaning of the problem better, but also clarifies the problem poser’s own mastery of concepts. As a result, the current study developed a competition-based problem-posing learning system (see Figure 2) to help students organize new and old knowledge, thereby constructing a more complete knowledge structure. Furthermore, the competition-based problem-posing feedback subsystem was developed to help students carry out higher-order thinking in the problem-posing learning process, and to achieve learning growth through problem revision and inquiry learning.

After students entered the learning system, they were guided to pose a question on their own, consisting of the stem and options. It would be stored in the problem-posing database after submission. The students obtained scores based on the depth of the posed questions. If the first question had great discrimination, the students were given a perfect score, indicating that the question and options were well designed. In contrast, if the posed question was too simple and lacked discrimination, the students would not get good scores. Competition encouraged students to be more cautious in formulating questions and options so that they would gain higher scores.

**Figure 3:**
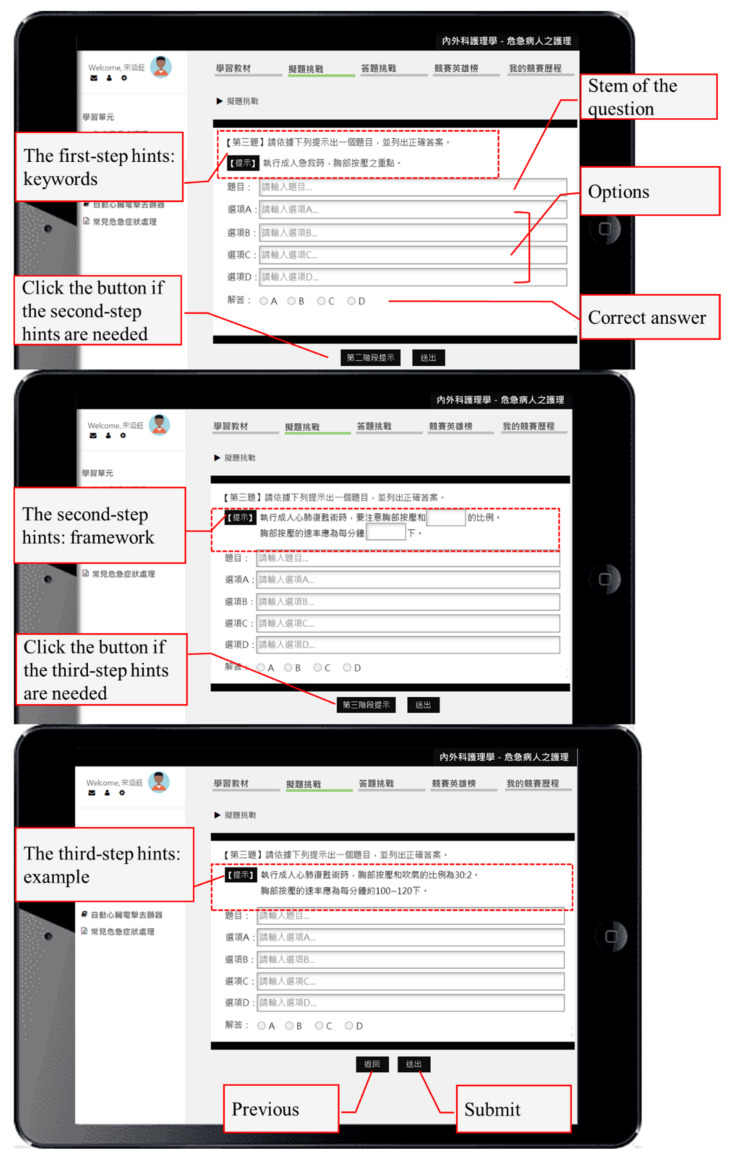
Three-step problem-posing-based learning scaffolding.

In the problem-posing process, the learning system provided an individual problem-posing-based learning scaffolding, as shown in Figure 3. Students could choose different steps of hints based on their needs. The competition-based problem-posing mobile learning system developed in this study provided three steps of hints. The first step was “keywords”: students were provided with keywords to realize the direction and scope of the question. The second step was “framework”: students were provided with an incomplete question to think about the question through filling in the blanks. The third step was “example”: students were provided with a complete question with the answer posed by an expert to think about, organize their knowledge, and then revise the question and answer. In this study, for example, the hint in the first step was “Please pose a question and four options, and mark the correct answer based on ‘the key points of chest compression in the first aid for adults’.” The hint in the second step was “Please pose a question and four options, and mark the correct answer based on ‘When performing cardiopulmonary resuscitation (CPR) for adults, be careful about the ratio of compression and _______. The rate of compression should be _______ per minute’.” The hint in the third step was “Please pose a question and four options, and mark the correct answer based on ‘When performing CPR for adults, the compression-ventilation ratio is 30:2. The compressions should be at a rate of 100 to 120 times a minute’.”

After posing a question, the students were guided to proceed with the competition. The system would record their answers according to their account ID and stored them in the learning portfolio database. The formula for calculating a score is:

(1-|accuracy rate-error rate|) * weighted ratio

Students’ posed questions would be answered by peers. A score for each question was calculated based on the accuracy rate and error rate, which was stored in the student account database to calculate their total scores instantly.

**Figure 4:**
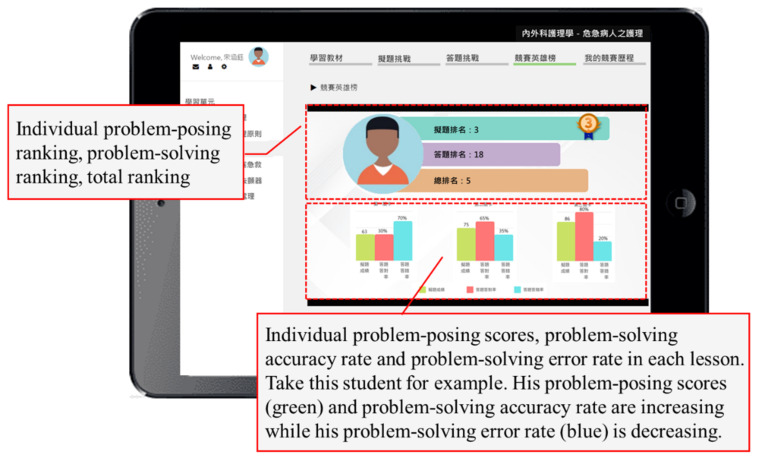
Leaderboard for the problem-posing competition.

The learning portfolio and management subsystem not only recorded students’ learning process and progress, but also analyzed their various performances based on the records (see Figure 4). These analytic results can serve as a reference for the teacher to improve the course content and learning activities, as well as for diagnosing students’ learning difficulties and providing personalized learning advice. The teacher was able to effectively manage and monitor students’ individual learning status from the learning portfolio module, and then adequately modify the instruction and conduct remedial teaching. In addition, the teacher could obtain the student learning diagnosis reports and related documents to grasp the learning situations of the class.

### 3.2. Participants and the Teaching Unit

This study was evaluated and approved by the research ethics committee of University. The participants were informed that participation in the study was voluntary. They were also informed of the details of the purpose of the study, the process, and how data would be collected and used. In addition, the participant data were treated as confidential. The data were coded before analysis to protect the privacy of the participants. Moreover, the data will be kept for 3 years; after that, the data will be destroyed following the regulations requested by the research ethics committee.

A quasi-experiment was conducted in the present study. A total of 96 students (males = 19, females = 77) from two classes at a university in northern Taiwan were recruited in this study. The participants were aged between 21 and 22. One class with 49 students was the experimental group, while the other class with 47 students was the control group. To avoid the two groups of students affecting each other, the classrooms of the two classes were located on different floors of the same building. In order to enhance the internal validity of this study, the variables which were irrelevant to the teaching experiment were controlled, including prior knowledge, instructor, teaching environment, learning content, and experiment period. All the participants were taught by the same teacher.

The teaching unit was Care of Critically Ill Patients. Apart from the school-based textbook, the teaching content also included domestic and foreign books and articles related to first aid training, as well as the training content from the Ministry of Health and Welfare, Taiwan. The unit of care of critically ill patients consisted of five lessons: procedures for critically ill patients, CPR, respiratory tract obstruction, automated external defibrillator (AED), and treatment of common emergencies.

### 3.3. Experimental Procedure

To explore the effectiveness and effects of the proposed learning approach, this study was conducted in the unit of care of critically ill patients at a university. The teaching activity was divided into two phases.

In the first phase, students carried out the learning activity with tablet computers in a specialized classroom. They received training for professional knowledge about care of critically ill patients and prior knowledge of basic skills for three weeks. In the specialized classroom, students could not only learn skills and practice, but also watch and learn repeatedly through tablet computers at the same time; their learning history was uploaded to the system through the Internet. At the end of the third week, students were administered a pre-test of their professional knowledge about the care of critically ill patients and a pre-questionnaire of their learning motivation. The pre-test aimed to explore the two groups’ prior knowledge before the learning activity, while the pre-questionnaire was used to examine their learning motivation before the experiment.

In the second phase, a learning activity was conducted in a computer classroom for students to pose and answer questions. The teacher explained and demonstrated how to operate the learning system for 30 min. Then, the experimental group adopted the competition-based problem-posing learning approach, while the control group adopted the conventional problem-posing learning system. Both groups had identical learning environments, learning materials, and the same teacher, and carried out the learning activity for three weeks (300 min in total). It should also be noted that the course was conducted in classrooms in a face-to-face mode. The students in both groups used smartphones to access the problem-posing mobile learning system. That is, both groups of students were guided by the three-step problem-posing-based learning scaffolding during the experiment. The only difference between the two groups was that the experimental group adopted the competition mode, while the control group did not.

**Figure 5:**
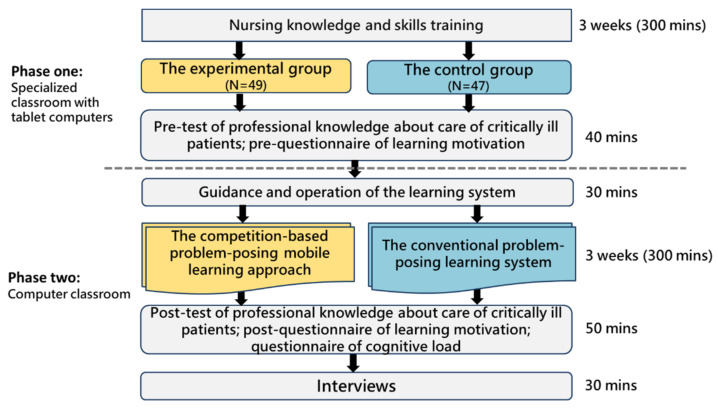
Experimental procedure.

After the experiment, both groups completed a post-test of their professional knowledge about care of critically ill patients, aiming to explore the effects of the competition-based problem-posing mobile learning approach on students’ cognitive performances. A post-questionnaire was administered to examine their learning motivation and cognitive load. In addition, the records of their learning behaviors and behavioral patterns were analyzed to corroborate the quantitative results. The learning portfolios not only recorded students’ learning process and progress, but also helped analyze their various performances. These analytic results can serve as a reference for the teacher to improve the course content and learning activity. Figure 5 shows the experimental procedure.

**Figure 6:**
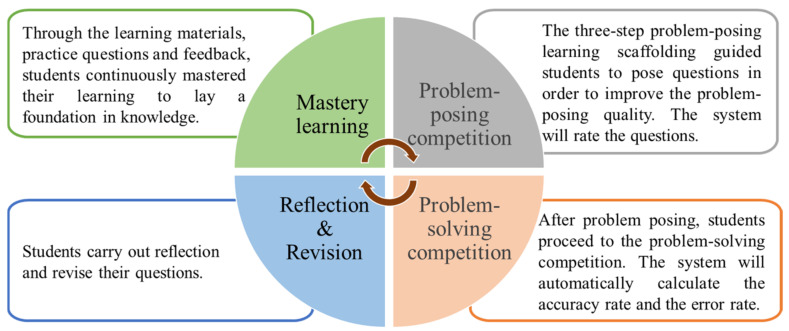
Procedure of the competition-based problem-posing mobile learning approach.

Figure 6 shows the competition-based problem-posing mobile learning approach. The competition-based problem-posing learning activity was a virtuous cycle. Through the learning materials, practice questions, and feedback, students continuously mastered their learning to lay a foundation for their knowledge. Next, the three-step problem-posing-based learning scaffolding instructed students to pose questions; it not only helped students to pose questions with higher quality, but also facilitated their learning performances and knowledge internalization. After posing questions, they proceeded to the problem-solving competition. During the problem-posing and problem-solving process, the system automatically calculated the accuracy rate and error rate. Students could view their own scores and the leaderboard at any time, carry out reflection, and revise their questions.

### 3.4. Measuring Tools

The learning achievement test, including the pre- and post-tests, were developed by a head nurse and a nurse practitioner with more than 10 years of nursing clinical working experience. The reliability was then verified by an expert group composed of faculty members in the nursing department, and the Rasch model was employed to examine its validity. Before the experiment, a pre-test was administered to understand students’ prior knowledge. The post-test was used to evaluate their learning achievement regarding care of critically ill patients. The pre- and post-tests contained 25 multiple-choice questions (75%) and two batteries of essay questions (25%), with a perfect score of 100. By applying the KR20 analysis, it was found that the reliability values of the pre-test and the post-test were 0.80 and 0.84, respectively. These values indicate that the tests were sufficiently reliable.

The learning motivation questionnaire was modified from Wang and Chen (2010) to explore students’ learning motivation before and after the learning activity. It consisted of six items with a 5-point Likert scale [50]. This questionnaire included two constructs, that is, intrinsic motivation (3 items) and extrinsic motivation (3 items). The Cronbach’s alpha values of the dimensions of intrinsic motivation and extrinsic motivation were 0.77 and 0.81, respectively. Intrinsic motivation refers to continuous or strengthened actions driven by a sense of satisfaction and happiness obtained in an activity. On the other hand, extrinsic motivation refers to continuous behaviors driven by external factors or rewards and punishments. The higher the score was, the higher intrinsic and extrinsic learning motivation a student had to engage in this experiment. The identical questionnaire was administered before and after the learning activity to examine the change in students’ intrinsic and extrinsic motivation.

The cognitive load questionnaire was adopted from Hwang, Yang, and Wang (2013). It consisted of eight items with a 5-point Likert scale. This questionnaire included two constructs, that is, mental load (5 items) and mental efforts (3 items) [37]. The Cronbach’s alpha values of the dimensions of mental load and mental efforts were 0.86 and 0.83, respectively. Mental load refers to the excessive load caused by the difficulty of materials or the challenging tasks. On the other hand, mental effort refers to the learning load resulting from the method, design, and materials used in a teaching activity. This questionnaire was administered after the learning activity, aiming to investigate the effects of the developed learning system on students’ learning.

### 3.5. Data Analysis

The collected data were analyzed using version 25 of the IBM SPSS Modeler software application. To understand if there was a significant difference for learning achievement and learning motivation between the experimental group and the control group, the ANCOVA was performed to analyze the post-test scores by excluding the effect of the pre-test scores. To meet the basic assumption of ANCOVA, the homogeneity of regression coefficients within groups was employed. To investigate if there was a significant difference in the cognitive load of the two groups, this study adopted the independent samples *t* test for the analysis. In addition to the quantitative results, we performed lag sequential analysis to calculate the frequency of each possible sequential pattern to explore the learning behaviors in the learning activity.

## 4. Results

### 4.1. Analysis of Learning Achievement

The homogeneity regression test showed that no significant difference between the pre-test scores of the two groups was found (*F* = 0.39, *p* = 0.154 > 0.05), indicating that the two groups had similar prior knowledge before the learning activity.

Table 1 shows the ANCOVA results of students’ learning achievements. The adjusted means and standard deviation of the experimental group were 87.60 and 1.88, while those of the control group were 79.92 and 1.82. The experimental group significantly outperformed the control group in terms of learning achievement (*F* = 9.05, *p* = 0.003 < 0.01, *η*^2^ = 0.09). The results specified that the competition-based problem-posing mobile learning approach was conducive to enhancing students’ learning achievement.

### 4.2. Analysis of Learning Motivation

The test of homogeneity regression showed that no significant difference between the pre-test scores of the two groups was found (*F* = 0.27, *p* = 0.61 > 0.05), implying that there was a consistent linear relationship between the two variables within the groups.

Table 2 shows the ANCOVA results of the students’ learning motivation. The adjusted means and standard deviation of the experimental group were 4.23 and 0.07, while those for the control group were 3.94 and 0.08. The experimental group had significantly higher post-test scores than the control group (*F* = 6.53, *p* = 0.012 < 0.05, *η*^2^ = 0.06). The results pointed out that the competition-based problem-posing mobile learning approach could increase students’ learning motivation.

### 4.3. Analysis of Cognitive Load

The *t*-test results are displayed in Table 3. It was found that the mean for the experimental group was 2.71, while that for the control group was 2.91. No significant difference was found for the cognitive load of the two groups (*t* = 1.49, *p* = 0.14 > 0.05), showing that the students had similar cognitive loads no matter which learning approach they adopted. Moreover, the means of the experimental group and the control group were lower than three, implying that the difficulty of the learning materials, adopted methods, and design of the learning activity in the two different approaches did not cause an excessive load for students.

### 4.4. Analysis of Learning Behaviors

To further examine the relationship between students’ learning behaviors and learning achievements during the learning process, we classified the possible learning behaviors into six categories, namely reading learning materials (A), posing questions (B), looking for the second-step hints (C), looking for the third-step hints (D), solving questions (E), and viewing learning portfolios (F). The detailed coding scheme for learning behaviors is illustrated in Table 4.

Table 5 shows the adjusted residuals table of the experimental group, among which 13 sequences were statistically meaningful, for example, A→A, A→B, A→C, and B→A. This sequential analysis was performed to calculate the frequency of one behavior connected to another. Then, the z-score was calculated. The formula for calculating the z-score is Z = (X − X)/s, with X as the frequency, X as the mean, and s as the standard deviation. If the z-score is greater than 1.96, it shows a significant difference in the sequence (*p* < 0.05), specifying a significant relationship between two behaviors.

**Figure 7:**
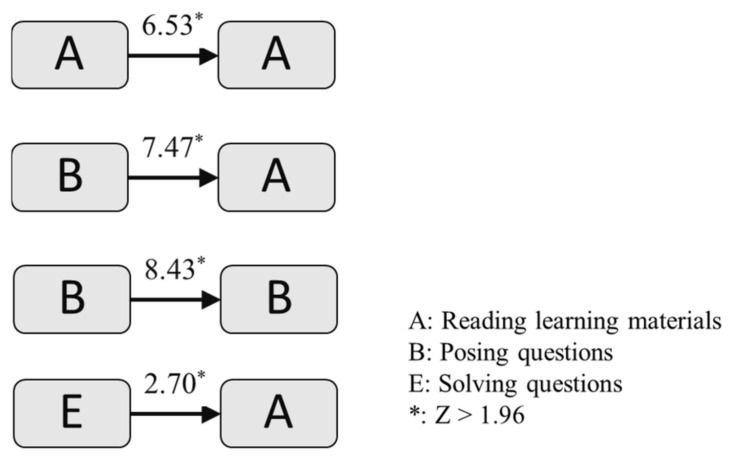
The simple behavioral transitional patterns.

We collected a total of 14,798 learning behaviors of the experimental group. Figure 7 displays the simple behavioral transitional patterns. It shows several significant simple patterns, for example, repeatedly reading the learning materials (A→A), reading the learning materials after posing questions (B→A), repeatedly posing questions (B→B), reading the learning materials to confirm the accuracy of their answers (E→A).

**Figure 8:**
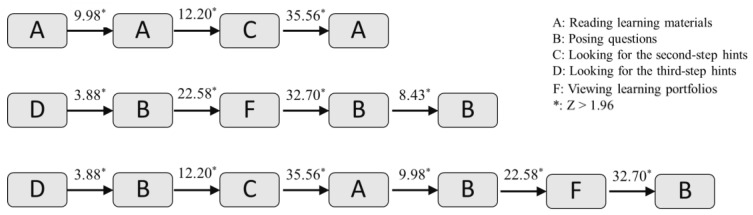
The complex behavioral transitional patterns.

Figure 8 shows the complex behavioral transitional patterns that are worth investigating. For instance, A→B→C→A indicated that after students read the learning materials, they started to pose questions. During problem-posing, after reading the first-step hints (keywords) provided by the system, students looked for the second-step hints (framework). Then, they read the learning materials again and completed problem-posing. This was an active learning behavioral sequence, showing that under the problem-posing-based learning scaffolding in the system, when students encountered problems, it encouraged them to look for hints or read learning materials for solutions. This not only cultivated their active learning but also promoted their problem-solving ability.

Another behavioral pattern, D→B→F→B→B, showed that students completed the problem-posing task after obtaining the third-step hints (example). They submitted the questions and then viewed their learning portfolios. Next, they looked back and reflected on their posed questions, revised them, and resubmitted them. It can be inferred from this sequence that the problem-posing-based learning scaffolding could help students actively look for answers and solve problems. In addition, competition in this study also played an important role, helping students to enhance their problem-posing quality. The underlying meaning was that students’ learning motivation and comprehension of knowledge were further improved. Since problem-posing was a higher-order thinking process, students were required to truly comprehend the content of the learning unit to pose questions with high quality.

Additionally, D→B→C→A→B→F→B denoted that the students conducted the problem-posing task after obtaining the third-step hints (example). During problem-posing, they looked for the first-step hints (keywords) and even the second-step hints (framework). Next, they also went back to read the learning materials. After completing the problem-posing task, they viewed their learning portfolios to understand the quality and process of their problem-posing, as well as their total scores and ranking. With the feedback, the students made revisions, carried out reflection, and submitted the modified questions.

Above all, the competition-based problem-posing mobile learning approach proposed in this study could promote students’ development of positive and active learning behaviors in the learning process. Unlike previous first aid training, in the competition-based problem-posing learning environment, the three-step problem-posing-based learning scaffolding (including keywords, framework, and examples) provided students with personalized learning aids in response to their current learning situation. It generated higher-order thinking by enabling students to internalize their acquired knowledge into the problem-posing task (including posing questions, listing options, and marking the correct answer). Furthermore, the present study incorporated competition and a leaderboard; students could view their scores and ranking, which successfully stimulated their thinking about problem-posing and problem-solving. According to the abovementioned learning behavioral sequence, after viewing their scores and ranking, the students would read the learning materials or use the hints in the system to increase their understanding. Then, they made revisions again to further enhance the quality of their questions. In the same vein, previous research has pointed out that problem-posing is a difficult task; students should have sufficient knowledge and understanding before posing questions.

As a result, based on the learning behavior sequences in this study, it was found that students not only completed the problem-posing task, but also actively looked for hints and read the learning materials during the process to clarify their misconceptions. The findings echoed the quantitative results of learning achievement, learning motivation, and cognitive load, indicating that the competition-based problem-posing mobile learning approach could effectively enhance students’ learning achievement and learning motivation, and did not cause an excessive cognitive load.

## 5. Discussion and Conclusions

The present study developed a competition-based problem-posing mobile learning approach and applied it in the unit of Care of Critically Ill Patients. This approach guided students to be familiar with the procedures and critical knowledge about care of critically ill patients, which facilitated their learning achievement and learning motivation, and reduced their cognitive load. This study integrated the three-step problem-posing-based learning scaffolding into the system to guide students’ problem-posing, that is, “keywords,” “framework,” and “example.” In addition, competition was incorporated to promote students to pose questions with higher quality, thereby effectively increasing their learning achievement and knowledge internalization.

In terms of learning achievement, the results specified that the experimental group adopting the competition-based problem-posing mobile learning approach significantly outperformed the control group adopting the conventional problem-posing learning system. With the three-step problem-posing learning scaffolding for both groups, competition could foster students to continuously learn new information during the process. To make sure whether their answers or decisions were accurate or not, students were more engaged in learning during the competition to clarify their misconceptions. This was consistent with Admiraal et al. (2011), who implied that students would be affected by scores, ranking, and rewards in a competition, and would then show competitive behaviors [19]. It was found that the more engaged students were in the competition, the more knowledge they acquired. In addition, based on the students’ learning portfolios, it was found that the learning behavior of reading the learning materials again after completing the problem-posing task occurred more than 500 times. This was different from the conventional teaching approach in which teachers had to constantly ask and give external stimulation for students to read the learning materials. In addition, once they had completed the problem-solving task, they would read the learning materials again to examine the accuracy of their answers. Interestingly, after viewing their personalized learning feedback, they made revisions, carried out reflections, and submitted the revised questions to improve their scores and ranking. According to the quantitative results and the analytic results of the learning portfolios, the reasons why the proposed competition-based problem-posing mobile learning approach was better than the conventional problem-posing learning system can be inferred. With the integration of competition and the leaderboard, students were allowed to view their scores and rankings, which successfully provoked them to think about their problem-posing and problem-solving methods [25], helped them to revise their questions again, and further enhanced the quality of their questions and their learning achievement [8,51].

With regard to learning motivation, the findings revealed that the experimental group adopting the competition-based problem-posing mobile learning approach significantly outperformed the control group adopting the conventional problem-posing learning system. These findings indicate that this approach was conducive to increasing the students’ learning motivation for the care of critically ill patients. For the experimental group, competition and the leaderboard were introduced in the learning system; students were able to view their scores and rankings, which encouraged them to improve their learning performances. Based on the analytic results of their learning portfolios, it was found that after completing the problem-posing task, students would view their learning portfolios to understand the quality and process of problem-posing, as well as their scores and ranking. Then, they went back to make a revision, carried out reflection, and submitted the revised questions. Previous studies have pinpointed that the elements of competition include scores or a leaderboard. Scores can assist students in evaluating their performances [31], while a leaderboard, a common mechanism, enhances students’ learning motivation and enables them to view the learning progress immediately [32,33]. Arikan and Ünal (2015) also disclosed that problem-posing ability would be affected by other factors such as learning motivation [47]. Competition enhanced the quality of students’ problem-posing. The underlying meaning was that students’ learning motivation and comprehension of knowledge were further improved. Since problem-posing was a higher-order thinking process, students were required to truly comprehend the content of the learning unit to pose high quality questions.

Regarding cognitive load, the independent sample *t*-test results uncovered that there was no significant difference between the competition-based problem-posing mobile learning approach and the conventional problem-posing learning system. The means of the two groups’ cognitive load were between 2.7 and 3.0 with a medium range, indicating that the difficulty of the teaching materials as well as the methods, design, and materials in the learning activity would not cause an excessive load for the students. Based on the interview results, students generally provided positive feedback on the three-step problem-posing-based learning scaffolding, for example, “During the problem-posing process, step-by-step hints provided by the system helped me understand the key points more and pose a good question,” “After completing the problem-posing task, I felt more confident in getting a better score next time,” “I had more confidence when encountering critically ill patients after this learning activity,” “This kind of learning activity enabled me to apply what I have acquired into practice,” and “This learning system enhanced my learning motivation and reduced my anxiety when encountering critically ill patients.” Although past research has shown that problem-posing can easily cause excessive cognitive load on students and reduce their learning motivation [13], this study found that with the appropriate scaffolding, providing moderately difficult challenges to students could effectively increase their learning achievement, learning performance, and learning motivation, thereby helping them put their acquired knowledge into practice.

This study proposed a competition-based problem-posing mobile learning approach, applied it to the unit of critically ill patients in a medical-surgical nursing course at a university, and explored its effects on the students. Integrated with problem-posing and competition, it was an innovative and worthwhile learning approach. Furthermore, the analysis of students’ learning concepts and problem-posing quality has recently become one of the important issues in educational practice. This study demonstrates its contribution and can serve as a reference for substantial studies and teaching practices related to mobile technology-assisted learning at home and abroad. In particular, it can be referred to in future clinical practice to enhance the quality and sustainability of the apprenticeship.

## Figures and Tables

**Table 1:** The ANCOVA results of students’ learning achievements.

Group	*N*	Mean	*SD*	Adjusted Mean	SE	*F*	*η* ^2^
Experimental Group	49	87.63	9.10	87.60	1.88	9.05 **	0.09
Control Group	47	79.89	15.25	79.92	1.82		

** *p* < 0.01.

**Table 2:** The ANCOVA results of students’ learning motivation.

Group	N	Mean	SD	Adjusted Mean	SE	*F*	*η^2^*
Experimental Group	49	4.22	0.53	4.23	0.07	6.53 *	0.06
Control Group	47	3.95	0.42	3.94	0.08		

* *p* < 0.05.

**Table 3:** The *t*-test results of students’ cognitive load.

Group	N	Mean	SD	*t*
Experimental Group	49	2.71	0.64	1.49
Control Group	47	2.91	0.62	

**Table 4:** The coding scheme of learning behaviors.

Code	Behavior	Record Point
A	Reading learning materials	Enter the learning materials
B	Posing questions	Start/submit the questions
C	Looking for the second-step hints	Click the button for the hints of the second step
D	Looking for the third-step hints	Click the button for the hints of the third step
E	Answering questions	Submit the solutions
F	Viewing learning portfolios	Entering the learning portfolio

**Table 5:** Adjusted residuals table of the experimental group.

Z-Score	A	B	C	D	E	F
A	6.53 *	9.98 *	33.59 *	−6.85	−10.17	−16.50
B	7.47 *	8.43 *	12.20 *	−8.57	−2.78	22.58 *
C	35.56 *	0.14	−1.55	−11.08	−10.55	−7.33
D	−11.21	3.88 *	−2.56	−40.18	−13.35	−0.93
E	2.07 *	−15.82	−1.60	−12.42	45.24 *	−12.24
F	−19.16	32.70 *	−12.08	−8.66	−14.16	65.91 *

Notes: * Z > 1.96; A: reading learning material; B: Posing questions; C: Looking for the second-step hints; D: Looking for the third-step hints; E: Answering questions; F: Viewing learning portfolios.

## Data Availability

No datasets were used in the present research.
